# Influence of low‐dose dopamine on exercise in fibrosing interstitial lung disease

**DOI:** 10.1113/EP093092

**Published:** 2026-03-26

**Authors:** Charlotte Chen, Jui‐Lin Fan, Michael Plunkett, Thalia Babbage, John Kolbe, Julian F. R. Paton, James P. Fisher

**Affiliations:** ^1^ Manaaki Mānawa – The Centre for Heart Research, Department of Physiology, Faculty of Medical & Health Sciences University of Auckland Auckland New Zealand; ^2^ Department of Medicine, Faculty of Medical & Health Sciences University of Auckland Auckland New Zealand

**Keywords:** chemoreflex, exercise, interstitial lung disease

## Abstract

Fibrotic interstitial lung disease (FILD) is associated with dyspnoea and exercise intolerance. In other cardiorespiratory conditions, heightened carotid body (CB) chemoreflex sensitivity is associated with reduced exercise capacity. We tested the hypothesis that CB chemoreflex inhibition would improve exercise endurance time (EET) and reduce dyspnoea in FILD. In this randomised, single‐blind study, 10 FILD patients (four women, 64 ± 8 years) completed the experimental procedures. Low‐dose dopamine infusion (2 µg/kg/min) was used to inhibit the CB chemoreflex, and its effectiveness verified by measuring the hypoxic ventilatory response. Participants performed two constant work rate cycle exercise tests (75% of peak work rate) to exhaustion with either dopamine or saline as control. Dopamine suppressed the CB chemoreflex sensitivity in seven of the ten participants. Overall, dopamine reduced chemoreflex sensitivity by 27% relative to control (−0.646 ± 0.344 vs. −0.472 ± 0.410 L/min/%, *P* = 0.103). EET (saline 460 ± 411 s vs. dopamine 363 ± 182 s, *P* = 0.305) and exertional dyspnoea scores were similar between the conditions. Among those displaying reduced CB sensitivity with dopamine, there was a positive correlation between the magnitude of reduction in sensitivity and improvement in EET (*r*_s_ = 0.943, *P* = 0.005). Dopamine was associated with reduced blood pressure and increased heart rate. Although low‐dose dopamine did not improve exercise capacity or dyspnoea in the group overall, subset analysis of those who achieved CB chemoreflex suppression (*n* = 7/10) with dopamine demonstrated a strong positive correlation between the reduction in sensitivity and EET improvement. This could suggest that the CB chemoreflex restrains exercise capacity in FILD.

## Abstract (highlights)

- **What is the central question of this study?**
  Does carotid body chemoreflex inhibition with low‐dose intravenous dopamine infusion improve exercise endurance time (EET) and reduce exertional dyspnoea in patients with fibrotic interstitial lung disease (FILD)?
- **What is the main finding and its importance?**
  Compared to saline infusion, mean EET and dyspnoea were unchanged with dopamine. However, in those who achieved CB chemoreflex suppression with dopamine, there was a strong positive correlation between the reduction in CB chemoreflex sensitivity and EET improvement. This could suggest that the CB chemoreflex restrains exercise capacity in FILD.

## 1 INTRODUCTION

Interstitial lung disease (ILD) encompasses a diverse array of conditions that result from inflammation and/or fibrosis of the lung parenchyma (Broaddus et al., 2016). Fibrosing ILD (FILD) is a subtype characterised by increased risk of progressive decline in lung function and early mortality (Cottin et al., 2019), although hypoxaemia generally manifests only in late‐stage disease. People with ILD commonly experience dyspnoea during physical activity (exertional dyspnoea) and reduced exercise tolerance (Collard & Pantilat, 2008; Holland, 2010). Exercise intolerance is associated with poor quality of life (Chang et al., 1999) and linked to mortality (Caminati et al., 2009; Fell et al., 2009; Lederer et al., 2006). In ILD, a key mechanism underlying these symptoms is ventilatory inefficiency, characterised by elevated minute ventilation (${\dot{V}}_{E}$) to the rate of CO_2_ production (${\dot{V}}_{CO_{2}}$) on cardiopulmonary tests (CPETs) (Faisal et al., 2016). This results from a combination of increased dead space ventilation due to mechanical constraints on tidal volume (*V*_T_), and elevated ventilatory drive secondary to increased afferent feedback (Phillips et al., 2020).

One important source of afferent feedback implicated in the control of breathing during exercise is that from carotid body (CB) chemoreceptors. Best known for mediating the hypoxic ventilatory response (HVR), the CB chemoreflex also plays a role in ventilatory control during normoxic conditions. CB chemoreflex activity at rest (tonicity) contributes to eupnoeic ventilatory drive (Croix et al., 1996; Dejours, 1962). During exercise, the CB chemoreflex contributes to the hyperventilation of heavy‐intensity exercise (Forster et al., 2012) and ‘fine‐tunes’ the hyperpnoea of mild to moderate exercise (Boetger & Ward, 1986; Griffiths et al., 1986; Whipp & Wasserman, 1980). The CB is also increasingly recognised as a contributor to sympathetic vasoconstrictor motor outflow and blood flow regulation during exercise in humans (Sayegh et al., 2024; Stickland et al., 2011, 2008). CB chemoreflex tonicity and sensitivity appear to be heightened in several chronic cardiorespiratory diseases including heart failure (Chua et al., 1996; Giannoni et al., 2009) and chronic obstructive pulmonary disease (COPD) (Phillips et al., 2018; Stickland et al., 2016). Augmented CB activity has been linked to an excessive ventilatory response to exercise (Chua et al., 1996; Plunkett et al., 2024) and exaggerated vasoconstriction in skeletal muscle (Sayegh et al., 2024; Stickland et al., 2007). Attenuating the CB chemoreflex in these conditions has been shown to enhance ventilatory efficiency, increasing exercise tolerance and alleviating exertional dyspnoea (Chua et al., 1997; Niewinski et al., 2017; Plunkett et al., 2024; Stulbarg & Winn, 1989). Notably, inhibition of CB activity with hyperoxia during whole‐body dynamic exercise led to improved exercise endurance time (EET) and lessened dyspnoea in ILD (Harris‐Eze et al., 1994; Schaeffer et al., 2017). However, a key limitation of these effects is the inability to differentiate the contributions of improved muscle oxidative metabolism (Kozlowski et al., 1971) from that of CB inhibition.

Herein, we investigated the effect of inhibition of the CB chemoreflex in patients with FILD. We used low‐dose intravenous dopamine (2 µg/kg/min), an established method to suppress the CB, which avoids the limitations associated with hyperoxia (Collins et al., 2020; Edgell et al., 2015; Janssen et al., 2009; Phillips et al., 2019). We tested the hypotheses that (1) inhibition of the CB chemoreflex would result in reduced exercise ventilation and dyspnoea, increasing EET, and (2) the magnitude of these effects would be greatest in those exhibiting the largest dopamine‐induced reductions in CB chemoreflex sensitivity.

## 2 METHODS

### 2.1 Ethical approval

This study was approved by the Healthy and Disability Ethics Committee, New Zealand (20/NTA/68) and prospectively registered in the Australian New Zealand Clinical Trials Registry (ACTRN12621000049875). Written informed consent was obtained from all participants following verbal and written explanation of the study procedures. The study was conducted according to the *Declaration of Helsinki* (2013).

### 2.2 Participants

Participants with stable FILD (no exacerbations in the preceding 6 weeks) were recruited from two respiratory clinics in Auckland, New Zealand. Eligibility criteria were clinical diagnosis of FILD, high‐resolution computed tomography evidence of pulmonary fibrosis (Sverzellati et al., 2015) and forced expiratory volume in 1 s/forced vital capacity ratio of >0.7. Exclusion criteria were greater than 15 pack‐year smoking history, emphysema on chest computed tomography, diagnosis of pulmonary sarcoidosis, regular use of inhaled bronchodilator treatment for airways disease, body mass index >35 kg/m^2^ and presence of significant comorbidities other than FILD that may contribute to dyspnoea and/or reduce exercise capacity.

All participants attended an initial visit during which they were familiarised with the study protocols. Anthropometric and general health information was collected. Activity‐related dyspnoea was assessed using Modified Medical Research Council Dyspnoea Scale (Fletcher et al., 1959) and health‐related quality of life assessed using King's Brief Interstitial Lung Disease questionnaire (KB‐ILD) (Patel et al., 2012). The participants’ most recent dynamic and static lung volumes (by body plethysmography) and test of gas transfer were obtained from medical records. During this visit participants performed a symptom‐limited, maximal incremental CPET to determine the maximal work rate (WR).

### 2.3 Experimental design

The study followed a randomised, single‐blind, placebo‐controlled crossover design. Participants refrained from caffeine, alcohol and exercise for 12 h, and adhered to a minimum 2‐h fasting period, prior to the experimental visits. A small‐bore intravenous catheter was inserted in the antecubital fossa/hand. Participants were seated in a comfortable position and instrumented. They wore a nose clip and were asked to breathe through a mouthpiece connected to a breathing circuit which was open to room air. Resting (pre‐infusion period) 5‐min cardiorespiratory data were collected. Following this, participants were administered acontinuous infusion of either dopamine 2 µg/kg/min (Niewinski et al., 2014; Sayegh et al., 2024; Stickland et al., 2011) (dopamine condition) or 0.9% saline (salinecondition); infusion rates were matched for dopamine and saline. After at leasta 10‐min wash‐in period (Boetger & Ward, 1986), participants underwent assessmentof the CB chemoreflex activity (see below). Finally, following a minimum 10‐minbreak, participants performed a constant WR CPET.

### 2.4 CB chemoreflex activity assessment

The ventilatory response to isocapnic hypoxia was used to evaluate the CB chemoreflex sensitivity. Participants wore a nose‐clip and breathed through a mouthpiece connected to the breathing circuit. The circuit was connected to a three‐way stopcock which allowed switching between ambient air and a customised rebreathing circuit. The circuit consisted of a two‐way non‐rebreathing valve, a second three‐way valve connected to a soda‐lime reservoir and a bypass, and 15 L rebreathing bag filled with room air. Following a 5‐min resting baseline data collection whilst breathing ambient air (‘post‐infusion’ period), the participant was switched to the rebreathing circuit at the end of a normal expiration. Isocapnia was maintain by manual regulation of airflow to the soda‐lime reservoir/bypass (Fan et al., 2011). The test was terminated when the end tidal partial pressure of O_2_ ($P_{\mathrm{ET}O_{2}}$) reached 45 mmHg or at the participant's request.

### 2.5 Cardiopulmonary exercise test

CPETs were performed on an electronically braked cycle ergometer (CORTEX bike M, CORTEX Biophysik, Leipzig, Germany) and in accordance with clinical exercise testing guidelines (American Thoracic Society & American College of Chest Physicians, 2003). Maximal incremental CPET (familiarisation visit) consisted of a 5‐min resting period, followed by a 2‐min warm‐up of unloaded pedalling, then 10 W/min increase in WR. Pedalling frequency was 60 rpm. Participants were provided with standardised verbal encouragement to exercise to exhaustion. Constant WR CPET (experimental visits) began in similar fashion, but unloaded pedalling was followed by an immediate increase in WR to 75% of peak WR. Participants were provided with verbal encouragement to exercise to exhaustion by an investigator who was blinded to the treatment. EET was recorded from the onset of loaded exercise to the point of exercise termination. All tests ended at the point of exhaustion or at the appearance of clinical indications for exercise termination (American Thoracic Society & American College of Chest Physicians, 2003).

### 2.6 Cardiorespiratory measures

For CB chemoreflex assessment, ventilatory flow (3830 Series, Heated Linear E Pneumotachometer; Hans Rudolph, Shawnee, KS, USA), end‐tidal partial pressure of CO_2_ ($P_{\mathrm{ETC}O_{2}}$) and $P_{\mathrm{ET}O_{2}}$ (Respiratory Gas Analyzer, ML206, ADInstruments, Bella Vista, NSW, Australia), peripheral saturation of O_2_ ($S_{pO_{2}}$, MLT321 and ML320/F, ADInstruments), heart rate (HR, 3‐lead electrocardiogram, BioAmp, FE231, ADInstruments), and finger blood pressure (BP, Human NIBP Nano interface, MLA382, ADInstruments) data were collected. Finger BP was validated with automated digital sphygmomanometer brachial artery BP measurements (Tango M2 BP monitor, SunTech, Morrisville, NC, USA). Due to technical reasons beat‐by‐beat BP was unavailable for one participant.

For CPETs, participants wore an oronasal mask (Hans Rudolph) attached to a spiroergometry system (Metalyzer 3B, CORTEX Biophysik). ${\dot{V}}_{E}$, *V*_T_, breathing frequency (*f*_R_), oxygen consumption (${\dot{V}}_{O_{2}}$), carbon dioxide production (${\dot{V}}_{CO_{2}}$) and respiratory exchange ratio (RER) were measured on a breath‐by‐breath basis. HR and rhythm were measured by 12‐lead electrocardiogram (custo cardio 300, custo med, Ottobrunn, Germany). BP was measured with a digital brachial artery sphygmomanometer (Tango M2 BP monitor, SunTech). $S_{pO_{2}}$ was monitored with finger pulse oximetry (WristOx2 Model 3150, Nonin Medical, Plymouth, MN, USA).

### 2.7 Symptom measures

Dyspnoea intensity and unpleasantness were assessed at rest, post‐infusion and at the termination of the isocapnic hypoxic test. During constant WR CPET, dyspnoea was assessed at the end of unloaded exercise, every 2 min during loaded exercise and at the end of exercise. Both intensity and unpleasantness were assessed using Borg's 0–10 category ratio scale (Borg, 1982) with phrasing according to methods previously described (Chen et al., 2025). At the end of exercise, participants were asked their reason for stopping and their rating of perceived exertion (RPE) obtained using the modified Borg RPE scale, with 0 corresponding to ‘no perceived exertion at all’ and 10 corresponding to ‘maximal perceived exertion’. A significant response was defined as ≥1 unit change in Borg score (Ries, 2005).

### 2.8 Data analysis

Baseline values for pre‐ and post‐infusion were taken as the average of beat‐to‐beat and breath‐by‐breath data over the last 2 min of the respective 5‐min periods. CB chemoreflex tonicity was estimated as Δ${\dot{V}}_{E}$ in response to dopamine infusion during air‐breathing at rest (${\dot{V}}_{E}$ post‐infusion minus ${\dot{V}}_{E}$ pre‐infusion). For CB chemoreflex sensitivity, breath‐by‐breath $S_{pO_{2}}$ from the isocapnic hypoxia period was bin‐averaged (3% bins) and plotted against ${\dot{V}}_{E}$. Sensitivity was calculated as the linear regression slope of ${\dot{V}}_{E}$ versus $S_{pO_{2}}$ (RStudio, v2023.09.1, Posit Software, Boston, MA, USA). CPET data were averaged over 15 s intervals prior to analysis. Unloaded and peak exercise was defined as the last 30 s of each exercise period. Ventilatory efficiency was estimated as: (1) nadir ${\dot{V}}_{E}/{\dot{V}}_{CO_{2}}$ and (2) ${\dot{V}}_{E}/{\dot{V}}_{CO_{2}}$ slope during loaded exercise (Phillips et al., 2020). The slope was measured by linear regression of ${\dot{V}}_{E}$ versus ${\dot{V}}_{CO_{2}}$ with exclusion of the non‐linear portion of the data (if present).

CB chemoreflex activation has a crucial role in cardiovascular control by regulating autonomic responses (Iturriaga et al., 2021; Zera et al., 2019). To provide an indication of resting cardiac autonomic control, spontaneous cardiac baroreflex sensitivity (cBRS) and heart rate variability (HRV) were evaluated using data from the post‐infusion period of both trials (saline vs. low‐dose dopamine). cBRS was calculated using the sequence technique (Parati et al., 2000) with CardioSeries software (v2.7, Ribeirão Preto, SP, Brazil). HRV was assessed using both time‐domain and frequency‐domain analyses (Kubios HRV Scientific, v 4.1.0, Kubios Oy, Kuopio, Finland). Time‐domain measures included the square root of the mean of the sum of successive differences in R‐R interval (RMSSD) and standard deviation of all normal sinus R‐R internals (SDNN). Fast Fourier transformation of R‐R variability was used for the frequency‐domain analysis, and the power spectra were quantified by the following: very low frequency power (0.0–0.04 Hz), low‐frequency power (LF; 0.04–0.15 Hz) and high‐frequency power (HF; 0.15–0.4 Hz) (Task Force of the European Society of Cardiology the North American Society of Pacing Electrophysiology, 1996).

### 2.9 Statistical analysis

Statistical analysis was performed using SPSS software, version 27 (IBM Corp., Armonk, NY, USA). Comparisons between dopamine and saline conditions were undertaken using Student's paired *t*‐test for normally distributed data and Wilcoxon's signed‐rank test for skewed data. Normality of residuals were verified by visualisation. For comparisons of constant WR CPET data, a relative isotime method was used (Nicolò et al., 2019). For each participant, the worst test (i.e. shorter EET) was used to identify four timepoints in which the two tests were segmented (25%, 50%, 75% and 100% of EET). The main effects of condition, time and their interaction were examined using two‐way ANOVA with repeated measures. *Post hoc* analysis, when appropriate, was carried out using Student's *t*‐test with a Bonferroni correction. In order to test the secondary hypothesis that the effect of dopamine on exercise capacity is related to the response in CB chemoreflex sensitivity, Spearman's rank correlation analysis was performed between sensitivity and key exercise variables. Values are presented as means ± standard deviation unless stated otherwise. A value of *P *< 0.05 was considered statistically significant. Where appropriate, effect sizes were calculated using Cohen's *d* with small, moderate and large effects being *d* ≥ 0.2, *d* ≥ 0.5 and *d* ≥ 0.8, respectively (Cohen, 1988).

## 3 RESULTS

### 3.1 Study participants

Eleven participants were recruited; one withdrew before completing all the visits. The demographic characteristics, baseline pulmonary function and incremental CPET responses of the 10 remaining participants are presented in Table 1. The most frequent diagnosis was connective tissue disease‐ILD (*n* = 5). Other diagnoses were idiopathic pulmonary fibrosis, vasculitis‐associated ILD, fibrotic non‐specific interstitial pneumonia, hypersensitivity pneumonitis and pulmonary fibrosis of unknown aetiology (*n* = 1 each). Pulmonary function measures of disease severity indicated moderate disease (forced vital capacity 68 ± 14%, diffusing capacity for carbon monoxide 57 ± 15%). However, daily activity dyspnoea (dyspnoea scale 1.0 ± 0.4) and quality of life (KB‐ILD 82 ± 10) were modestly affected. During incremental CPET, the ${\dot{V}}_{E}/{\dot{V}}_{CO_{2}}$ nadir was 30 ± 2 and ${\dot{V}}_{E}/{\dot{V}}_{CO_{2}}$ slope was 34 ± 5. Co‐morbidities and long‐term prescription medications are presented in the Appendix (Tables A1 and A2).

**TABLE 1:** Participant demographics, pulmonary function and incremental CPET responses.

Parameter	Value
Age (years)	64 ± 8
Female, *n* (%)	4 (40)
Weight (kg)	81.2 ± 8.4
Height (m)	1.74 ± 0.08
BMI (kg/m^2^)	30.1 ± 3.1
Smoking (pack years)	2.0 ± 4.4
MRC dyspnoea scale	1.0 ± 0.4
KB‐ILD score	82.2 ± 9.9
Pulmonary function	
FVC (L)	2.58 ± 0.83
FVC (% pred)	67.8 ± 14.1
TLC (% pred)	71.5 ± 10.5
DLCO (% pred)	56.5 ± 15
Incremental CPET responses at peak exercise	
WR (W)	89 ± 36
${\dot{V}}_{O_{2}}$ (L/min)	1.31 ± 0.48
${\dot{V}}_{O_{2}}$/kg (mL/min/kg)	15.87 ± 4.59
${\dot{V}}_{E}$ (L/min)	51.52 ± 20.14
*f*_R_ (breaths/min)	36 ± 6
*V*_T_ (L)	1.43 ± 0.45
$S_{pO_{2}}$ (%)	92 ± 4
Breathing reserve (%)	29 ± 23
RER	1.01 ± 0.06
${\dot{V}}_{E}/{\dot{V}}_{CO_{2}}$	33.69 ± 3.70
HR (beats/min)	119 ± 15
HR reserve (%)	32 ± 25
SBP (mmHg)	169 ± 22
DBP (mmHg)	84 ± 7

Abbreviations: BMI, body mass index; CPET, cardiopulmonary exercise test; DBP, diastolic blood pressure; DLCO, diffusing capacity for carbon monoxide; *f*_R_, breathing frequency; FVC, forced vital capacity; HR, heart rate; KB‐ILD, King's Brief Interstitial Lung Disease questionnaire; MRC, modified Medical Research Council; RER, respiratory exchange ratio; SBP, systolic blood pressure; $S_{pO_{2}}$, peripheral oxygen saturation; TLC, total lung capacity; ${\dot{V}}_{CO_{2}}$, carbon dioxide production; ${\dot{V}}_{E}$, minute ventilation; ${\dot{V}}_{O_{2}}$, oxygen consumption; *V*_T_, tidal volume; WR, work rate.

### 3.2 CB chemoreflex activity

**FIGURE 1:**
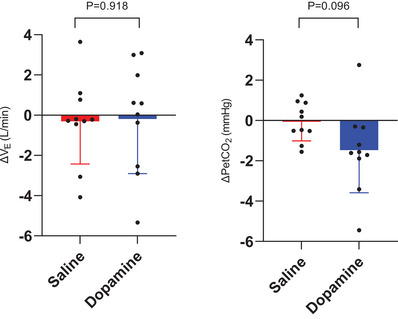
Resting change in ventilation and end‐tidal partial pressure of CO_2_ (pre‐infusion minus post‐infusion) in saline and dopamine trials. Bars are means ± standard deviation. *n* = 10 for both saline and dopamine conditions. Paired *t*‐test. Δ$P_{\mathrm{ETC}O_{2}}$, change in end‐tidal partial pressure of CO_2_; Δ${\dot{V}}_{E}$, change in minute ventilation.

Table 2 summarises the resting cardiorespiratory variables for the pre‐infusion and post‐infusion periods. There was a trend towards a rise in $P_{\mathrm{ETC}O_{2}}$ (*d* = 0.42) and reduction in $P_{\mathrm{ET}O_{2}}$ (*d* = 0.81) following dopamine. The infusion‐induced Δ${\dot{V}}_{E}$ (post‐infusion minus pre‐infusion) was −0.31 ± 2.12 and −0.18 ± 2.72 L/min for the saline and dopamine trials, respectively (*P* = 0.918, Figure 1).

**TABLE 2:** Cardiorespiratory variables before and after start of infusion.

	Saline trial			Dopamine trial		
	Pre‐infusion	Post‐infusion	*P*	Pre‐infusion	Post‐infusion	*P*
Respiratory parameters						
${\dot{V}}_{E}$ (L/min)	14.24 ± 4.24	14.55 ± 3.52	0.657	13.29 ± 3.13	13.48 ± 3.61	0.835
*f*_R_ (breaths/min)	19 ± 7	19 ± 7	0.691	18 ± 8	18 ± 6	0.915
*V*_T_ (L)	0.79 ± 0.15	0.81 ± 0.15	0.661	0.80 ± 0.20	0.79 ± 0.15	0.727
$S_{pO_{2}}$ (%)	96 ± 2	96 ± 2*	0.520	96 ± 2	95 ± 2*	0.002
$P_{\mathrm{ET}O_{2}}$ (mmHg)	98.0 ± 4.5	96 ± 4.7*	0.193	97.1 ± 6.3	92.5 ± 5.0*	0.051
$P_{\mathrm{ETC}O_{2}}$ (mmHg)	43.0 ± 2.3	43.1 ± 2.0	0.843	42.3 ± 3.9	43.8 ± 3.3	0.055
Dyspnoea intensity	0.5 ± 0.7	0.5 ± 0.8	1.000	0.5 ± 1.0	0.5 ± 1.0	
Dyspnoea unpleasantness	0.3 ± 0.4	0.4 ± 0.7	0.343	0.4 ± 0.7	0.4 ± 0.7	
Cardiovascular parameters						
HR (beats/min)	69 ± 12	66 ± 13	0.225	69 ± 15	71 ± 13	0.143
SBP (mmHg)	131 ± 18	137 ± 20*	0.022	126 ± 13	122 ± 14*	0.422
DBP (mmHg)	81 ± 10	80 ± 10	0.305	81 ± 9	74 ± 10	0.031
MAP (mmHg)	98 ± 12	99 ± 13	0.077	96 ± 9	90 ± 8	0.100

*n* = 9 for SBP, DBP and MAP. *P < 0.05 post‐infusion values between conditions. Pre‐infusion values between conditions were all *P *> 0.05. Abbreviations: DBP, diastolic blood pressure; *f*_R_, breathing frequency; HR, heart rate; MAP, mean arterial pressure; $P_{\mathrm{ETC}O_{2}}$, end tidal partial pressure of carbon dioxide; $P_{\mathrm{ET}O_{2}}$, end tidal partial pressure of oxygen; SBP, systolic blood pressure; $S_{pO_{2}}$, peripheral oxygen saturation; ${\dot{V}}_{E}$, minute ventilation; *V*_T_, tidal volume.

**FIGURE 2:**
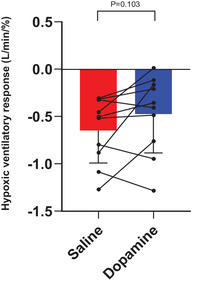
Hypoxic ventilatory responses during saline and dopamine infusion. A more negative value is equivalent to a higher chemoreflex sensitivity. *n* = 10 for both saline and dopamine conditions. Histograms are mean ± standard deviation. Paired *t*‐test.

Dopamine suppressed CB chemoreflex sensitivity in seven (of ten) participants (Figure 2). The overall sensitivity was −0.646 ± 0.344 L/min/% for saline and −0.472 ± 0.410 L/min/% for dopamine (*P* = 0.103). There was no significant correlation between baseline (saline) sensitivity and pulmonary function measures of disease severity. Dyspnoea ratings at the end of isocapnic hypoxia were similar between conditions (saline vs. dopamine; intensity: 2.3 ± 1.9 vs. 2.5 ± 2.9 *P* = 0.843, unpleasantness: 1.9 ± 1.4 vs. 2.1 ± 2.4 *P* = 0.751). cBRS and HRV were not different between the saline and dopamine trials (Table 3).

**TABLE 3:** Cardiac baroreflex sensitivity and heart rate variability during post‐infusion periods.

	Saline	Dopamine	*P*
cBRS			
Gain (ms mmHg^−1^)	9.98 ± 11.38	10.36 ± 9.25	0.595
Number of sequences (*n*)	9 ± 7	18 ± 20	0.144
BEI	0.22 ± 0.16	0.41 ± 0.27	0.099
HRV			
RMSSD (ms)	39.4 ± 27.1	31.9 ± 19.1	0.087
SDNN (ms)	30.9 ± 17.3	27.9 ± 14.8	0.397
HF (ms^2^)	689 ± 1293	446 ± 682	0.315
LF (ms^2^)	270 ± 239	216 ± 245	0.589
TP (ms^2^)	990 ± 1410	711 ± 991	0.139
HF (n.u.)	58 ± 17	63 ± 11	0.428
LF (n.u.)	42 ± 17	37 ± 11	0.421
LF/HF ratio	0.88 ± 0.57	0.64 ± 0.31	0.287

For cBRS variables *n* = 8. For HRV variables *n* = 9. BEI, baroreflex effectiveness index; HF, high frequency; LF, low frequency; n.u., normalised units; RMSSD, square root of the mean of the sum of successive differences in R–R interval; SDNN, standard deviation of all normal sinus R–R intervals; TP, total power.

### 3.3 Constant WR CPET

**FIGURE 3:**
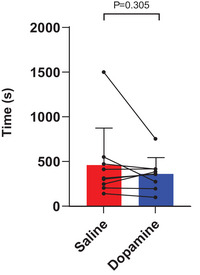
Exercise endurance time during constant WR CPET in saline and dopamine trials. *n* = 9 for both saline and dopamine conditions. Bars are means ± standard deviation. Paired *t*‐test.

**FIGURE 4:**
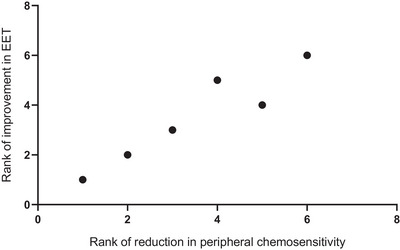
Spearman's rank correlation between reduction in peripheral chemosensitivity and improvement in exercise endurance time (EET) in the subset of participants with carotid body chemoreflex suppression by dopamine. *n* = 6, as EET was not available in one patient due to premature termination of exercise. Improvement in EET: dopamine condition EET minus control condition EET. Reduction in peripheral chemosensitivity: absolute value of dopamine condition chemosensitivity minus control condition chemosensitivity. Rank 1 refers to the greatest reduction in chemosensitivity/improvement in EET.

CPET was prematurely terminated in one participant due to a reduction in systolic BP of >20 mmHg during exercise. This participant was excluded in analyses involving EET, but their exercise data were retained in other analyses. In the remaining nine participants, dopamine did not improve the EET compared to saline (saline 460 ± 411 s vs. dopamine 363 ± 182 s, *P* = 0.305, Figure 3). There was a non‐significant moderate positive correlation between the magnitude of reduction in chemoreflex sensitivity and improvement in EET (*r*_s_ = 0.617, *P* = 0.077). Subset analysis limited to participants with CB chemoreflex suppression by dopamine (*n* = 6) also demonstrated no improvement in EET with dopamine. However, the correlation between the magnitude of reduction in sensitivity and improvement in EET was strongly positive (*r*_s_ = 0.943, *P* = 0.005, Figure 4).

During unloaded exercise, dopamine reduced ${\dot{V}}_{E}$ (*d* = 0.62) and *f*_R_ (*d* = 0.49) compared to saline (Table 4). All BP measures were reduced with dopamine with a concomitant increase in HR. Peak ${\dot{V}}_{O_{2}}$ attained was similar between conditions. RPE at peak exercise was 5.3 ± 2.4 and 5.8 ± 2.1 for the saline and dopamine trials, respectively (*P* = 0.575).

**TABLE 4:** Cardiorespiratory responses during constant WR CPET.

	Unloaded exercise			Peak exercise		
	Placebo	Dopamine	*P*	Placebo	Dopamine	*P*
WR (W)	0	0		69 ± 30	69 ± 30	
${\dot{V}}_{O_{2}}$ (L/min)	0.49 ± 0.06	0.47 ± 0.06	0.265	1.25 ± 0.43	1.27 ± 0.44	0.586
${\dot{V}}_{O_{2}}$/kg (mL/min/kg)	6.06 ± 0.93	5.81 ± 0.93	0.264	15.22 ± 4.00	15.41 ± 4.11	0.638
RER	0.82 ± 0.04	0.82 ± 0.04	0.891	1.00 ± 0.05	1.02 ± 0.08	0.252
${\dot{V}}_{E}$ (L/min)	16.88 ± 3.56	14.91 ± 2.73	0.009	50.28 ± 19.00	50.02 ± 17.78	0.919
*f*_R_ (breaths/min)	23 ± 6.21	20 ± 6	0.031	36 ± 7	35 ± 6	0.750
*V*_T_ (L)	0.78 ± 0.17	0.76 ± 0.12	0.586	1.39 ± 0.38	1.41 ± 0.45	0.675
$S_{pO_{2}}$ (%)	96 ± 2	95 ± 3	0.545	93 ± 4	92 ± 4	0.129
${\dot{V}}_{E}/{\dot{V}}_{CO_{2}}$	34.16 ± 4.69	31.75 ± 3.64	0.004	35.56 ± 2.64	34.42 ± 4.35	0.473
$P_{\mathrm{ETC}O_{2}}$ (mmHg)	34.1 ± 2.1	36.6 ± 2.9	0.002	34.0 ± 4.0	35.0 ± 4.1	0.482
$P_{\mathrm{ET}O_{2}}$ (mmHg)	109.3 ± 3.06	105.0 ± 5.8	0.022	116.6 ± 3.8	116.5 ± 5.1	0.950
HR (beats/min)	77 ± 14	85 ± 14	0.013	117 ± 14	130 ± 14	0.019
O_2_ pulse (mL/beat)	6.49 ± 1.27	5.75 ± 1.31	0.085	10.72 ± 3.50	9.71 ± 2.99	0.061
SBP (mmHg)	142 ± 27	122 ± 22	0.043	174 ± 30	169 ± 22	0.548
DBP (mmHg)	86 ± 17	72 ± 10	0.006	93 ± 11	77 ± 7	0.003
MAP (mmHg)	105 ± 19	88 ± 11	0.013	120 ± 12	108 ± 10	0.007

*n* = 7 saline unloaded SBP, DBP, MAP. *n* = 9 peak SBP, DBP, MAP. Abbreviations: DBP, diastolic blood pressure; *f*_R_, breathing frequency; HR, heart rate; MAP, mean arterial pressure; $P_{\mathrm{ETC}O_{2}}$, end‐tidal partial pressure of carbon dioxide; $P_{\mathrm{ET}O_{2}}$, end‐tidal partial pressure of oxygen; RER, respiratory exchange ratio; SBP, systolic blood pressure; $S_{pO_{2}}$, peripheral oxygen saturation; ${\dot{V}}_{E}$, minute ventilation; ${\dot{V}}_{O_{2}}$, oxygen consumption; *V*_T_, tidal volume; WR, work rate.

**FIGURE 5:**
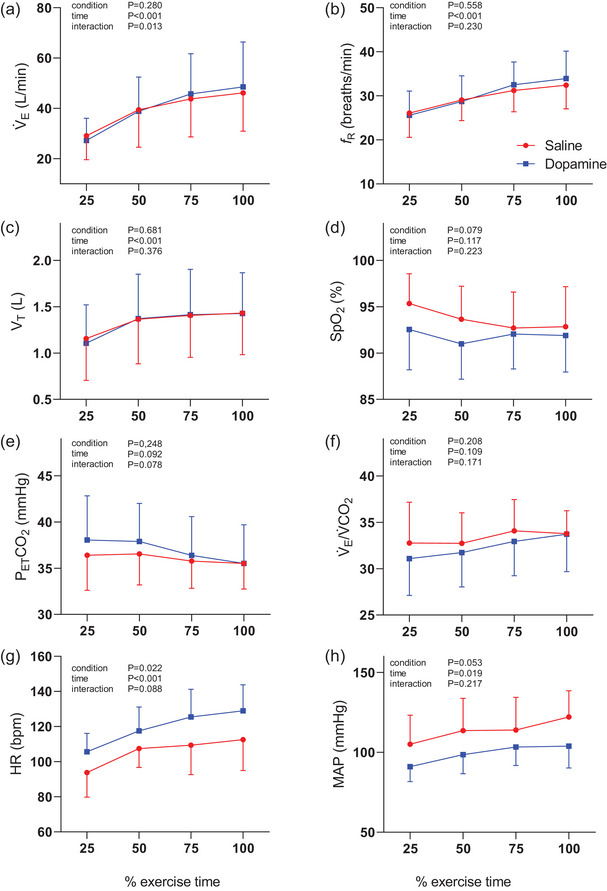
Respiratory and cardiovascular responses during the loaded portion of constant WR CPET in saline and dopamine trials. (a) Minute ventilation (${\dot{V}}_{E}$); (b) respiratory frequency (*f*_R_); (c) tidal volume (*V*_T_); (d) peripheral oxygen saturation ($S_{pO_{2}}$); (e) end‐tidal partial pressure of carbon dioxide ($P_{\mathrm{ETC}O_{2}}$); (f) ventilatory equivalent for carbon dioxide (${\dot{V}}_{E}/{\dot{V}}_{CO_{2}}$), (g) heart rate (HR); (h) mean arterial pressure (MAP). *n* = 9 in both dopamine and saline conditions. Data are means ± standard deviation. Two‐way ANOVA with repeated measures.

Oxygen uptake (${\dot{V}}_{O_{2}}$/kg) was similar between conditions throughout loaded exercise (condition *P* = 0.264, time *P *< 0.001, interaction *P* = 0.095). The cardiorespiratory responses to loaded exercise are presented in Figure 5. There was an interaction between treatment and time for ${\dot{V}}_{E}$ (Figure 5a), but *post hoc* testing did not demonstrate a significant difference at any of the time points. Analysis limited to the subset of those who responded to dopamine (*n* = 7) also did not reveal a difference in ${\dot{V}}_{E}$ (condition *P* = 0.894, time *P *< 0.001, interaction *P* = 0.064). The nadir ${\dot{V}}_{E}/{\dot{V}}_{CO_{2}}$ was 30 ± 2 and 29 ± 3 for the saline and dopamine trials, respectively (*P* = 0.379). The mean slope of ${\dot{V}}_{E}/{\dot{V}}_{CO_{2}}$ was also similar between the conditions (saline 34 ± 4 vs. dopamine 34 ± 6, *P* = 0.940).

**FIGURE 6:**
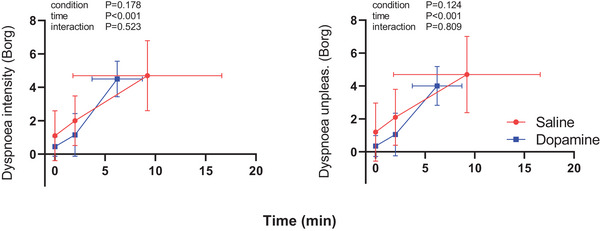
Dyspnoea scores at unloaded exercise, 2‐min isotime and peak exercise during constant WR CPET. *n* = 9 in both dopamine and saline conditions. Data are means ± standard deviation. Two‐way ANOVA with repeated measures.

Dopamine did not alter the dyspnoea intensity or unpleasantness ratings during exercise in the group overall (Figure 6) nor in the subset of responders (not shown). The rate of dyspnoea intensity increase, estimated by the linear regression slope of intensity rating between unloaded exercise and peak exercise was higher in the dopamine trial (saline 0.54 ± 0.35 units/min vs. dopamine 0.73 ± 0.26 units/min, *P* = 0.013). For both conditions, the predominant reason for termination of exercise was leg fatigue (saline *n* = 5, dopamine *n* = 6). At the end of exercise, the reported leg fatigue was not different (saline 5.7 ± 1.8 vs. dopamine 6.4 ± 1.7, *P* = 0.360).

## 4 DISCUSSION

The aim of this study was to determine whether suppression of the CB chemoreflex using intravenous low‐dose dopamine could modify the cardiorespiratory response to whole‐body dynamic exercise and reduce dyspnoea in patients with FILD. The major findings are: (1) dopamine did not extend the EET, (2) there was evidence of hypoventilation with dopamine at rest and during unloaded exercise, (3) there was no evidence of reduced ${\dot{V}}_{E}$ or improved ventilatory efficiency during loaded exercise, and (4) there was no improvement in dyspnoea with dopamine. In our sub‐analysis, we found a strong correlation between the magnitude of reduction in chemoreflex sensitivity and improvement in EET, whilst other key outcomes were not different between the two conditions. Of note, in this population dopamine was associated with significant haemodynamic changes.

Historically, the CB has been considered to play a minor role in the cardiorespiratory response to exercise compared with its well‐established hypoxia‐mediated reflexes (Boetger & Ward, 1986; Kaufman & Forster, 1996; Stickland et al., 2007). Although its involvement in sympathetic‐mediated blood flow redistribution has been demonstrated (Stickland et al., 2011, 2008), uncertainty has remained regarding its overall clinical significance and the mechanism(s) underlying CB activation during exercise (Forster et al., 2012). More recent studies, however, have provided new insight. Collins et al. (2025) performed a secondary analysis of pooled data from two randomised placebo‐controlled trials in patients with chronic heart failure and COPD, and healthy controls. The studies had individually shown no effect of CB inhibition (using low‐dose dopamine) on EET. They found that regardless of disease condition, those with high HVR demonstrated significant improvement in EET with CB inhibition. This is consistent with results of an earlier study in pulmonary arterial hypertension (Plunkett et al., 2024). Notably, in the pooled analysis the improvement in EET was associated with enhanced vascular conductance and reduced leg discomfort, demonstrating for the first time that CB‐mediated restraint of blood flow is sufficient to influence exercise tolerance in humans (Collins et al., 2025). Findings from Andrade et al. (2025) showed that in a rat model, CB glomus cells are critical in regulating peak oxygen consumption during physical exertion through lactate sensing (Andrade et al., 2025). The present study is therefore situated within this emerging framework.

Dopamine exerts an inhibitory effect on the CB via dopamine D2 receptors on the glomus cells (González et al., 1995). There appears to be significant interindividual variability in CB sensitivity to dopamine (Limberg et al., 2016). Dopamine infusion at 2 µg/kg/min has been shown to inhibit the CB chemoreflex in humans (Niewinski et al., 2014; Sayegh et al., 2024; Stickland et al., 2011), while avoiding the potential for significant adrenergic stimulation at higher doses (Collins et al., 2020; Fisher et al., 2018; Phillips et al., 2018). Even though the difference in chemoreflex sensitivity between saline and dopamine conditions was not statistically significant (*P* = 0.103), infusion of dopamine resulted in a 27% reduction in chemosensitivity. A further suggestion of CB engagement was the observed trend towards a rise in $P_{\mathrm{ETC}O_{2}}$ and reduction in $P_{\mathrm{ET}O_{2}}$ (with large effect size) following dopamine infusion at rest (Table 2), indicative of relative hypoventilation. In addition, ${\dot{V}}_{E}$ was reduced with dopamine during unloaded exercise. Collectively, our data indicate that dopamine infusion exerted an inhibitory effect on breathing at rest and during unloaded exercise.

### 4.1 CB chemoreflex activity

Unlike that in heart failure and COPD (Edgell et al., 2015; Phillips et al., 2019), we did not find evidence of increased CB chemoreflex tonicity in FILD (Figure 1). We decided a priori not to include a healthy control group, as it was considered more meaningful to determine whether CB chemoreflex inhibition would improve EET and dyspnoea, rather than comparing the differences in the effect between FILD and healthy controls. As such, no direct conclusions can be drawn regarding whether FILD is associated with heightened CB chemoreflex sensitivity. Nevertheless, data from other studies indicate that the CB chemosensitivity for healthy individuals of similar ages to our cohort ranges from −0.07 to −0.21 L/min/% (Edgell et al., 2015; Phillips et al., 2019), lower than that found in our FILD patients. Whilst differences in hypoxia protocol limit direct comparisons, there is evidence that progressive versus steady‐state protocols do not have a significant impact on HVR values (Oeung et al., 2023). Therefore, our findings raise the possibility that CB chemoreflex sensitivity may be elevated in patients with FILD.

### 4.2 Ventilation and ventilatory efficiency

Ventilatory inefficiency, reflected by an elevated ${\dot{V}}_{E}/{\dot{V}}_{CO_{2}}$ relationship, is linked to reduced exercise tolerance and increased dyspnoea. In heart failure and pulmonary hypertension, attenuation of CB chemoreflex activity has been associated with enhanced ventilatory efficiency (Chua et al., 1997; Niewinski et al., 2017; Plunkett et al., 2024). When less ventilation is required for a given CO_2_ output, exercise tolerance and dyspnoea improves through reduced work of breathing and delayed onset of ventilatory limitation (Sietsema et al., 2020). In ILD, impaired ventilatory efficiency is both common and associated with poor survival (Barratt et al., 2020). As expected, our participants demonstrated an elevated ${\dot{V}}_{E}/{\dot{V}}_{CO_{2}}$ slope of 34 (normal < 31; Phillips et al., 2020); however, dopamine was not associated with improvement in the slope, nadir nor peak exercise ${\dot{V}}_{E}/{\dot{V}}_{CO_{2}}$. There was no indication that dopamine altered the pattern of ventilation, which in ILD is characterised by shallow and rapid breathing, contributing to increased dead space ventilation (Molgat‐Seon et al., 2019). If CB chemoreflex inhibition had reduced ventilatory drive, then we would expect to observe reduced ${\dot{V}}_{E}$ with evidence of relative hypoventilation (e.g. elevated $P_{\mathrm{ETC}O_{2}}$). This was present at unloaded but not loaded exercise. Unfortunately, the only previous ILD studies of hyperoxia during exercise that reported ventilatory efficiency was a pilot study (*n* = 6), where peak exercise ${\dot{V}}_{E}/{\dot{V}}_{CO_{2}}$ was unchanged (Cournoyer et al., 2020). In a larger study (*n* = 20) hyperoxia resulted in reduction in ${\dot{V}}_{E}$ and increased $P_{\mathrm{ETC}O_{2}}$ at iso‐time and peak exercise compared to room air (Schaeffer et al., 2017). While suggestive of reduced ventilatory drive, it is important to bear in mind that hyperoxia also reduces ${\dot{V}}_{CO_{2}}$ and RER likely through metabolic mechanisms (Howley et al., 1983).

In contrast to loaded exercise, during unloaded exercise dopamine was associated with a reduction in ${\dot{V}}_{E}$ of moderate effect size (*d* = 0.62), predominantly mediated by decreased *f*_R_ (Table 4). The concomitant rise in $P_{\mathrm{ETC}O_{2}}$ suggests relative hypoventilation mediated by reduced ventilatory drive, rather than an improvement in the matching of ventilation to metabolic demand. This observation is potentially clinically relevant. The oxygen uptake response to unloaded cycling is comparable to slow‐walking (Porszasz et al., 2003). Therefore, this reduction in ventilation may be more relevant to activities of daily living in individuals with severe exercise tolerance impairment. In addition, the reduction in *f*_R_ may counteract the shallow tachypnoeic breathing pattern observed in ILD.

Despite the potential clinical implications, this finding raises several important questions. First, ventilation tends to be more variable at both rest and during lower intensity exercise, and therefore we cannot rule out the possibility that the observation is spurious. Second, the reason for the absence of sustained hypoventilation during loaded exercise remains unclear. CB afferents modulate the ventilatory kinetics during non‐steady state exercise (Griffiths et al., 1986; Wasserman et al., 1975), and indeed low‐dose dopamine inhibition of the CB slowed the rise in ${\dot{V}}_{E}$ in healthy humans (Boetger & Ward, 1986). Steady state is unlikely to be achieved when exercising at 75% of peak WR. In addition, the CB are widely accepted to contribute to the hyperventilation of heavy exercise (Forster et al., 2012).

The lack of sustained reduction in ${\dot{V}}_{E}$ during loaded exercise with CB inhibition may reflect the overlapping mechanisms involved in exercise hyperpnoea. When one mechanism is attenuated, others compensate to maintain exercise ventilation (Forster et al., 2012; Ward, 2019). We speculate that in FILD, elevated central command may contribute to the observed findings. Indirect evidence suggests that onset of high‐intensity, fatiguing exercise is associated with a curvilinear increase in feedforward central command (Mateika & Duffin, 1994). Therefore, it has been hypothesised that during heavy exercise, as locomotor muscle fatigue occurs, more central command is required to maintain muscle force (Forster et al., 2012). It is possible that in the FILD patients, heightened muscle fatigue provided augmented central command, which served as additional input for ventilatory drive when CB afferent feedback was reduced. This is supported by previously published data demonstrating skeletal muscle dysfunction in FILD. Compared to the healthy population, FILD is associated with both reduced muscle strength (Nishiyama et al., 2005; Watanabe et al., 2013) and reduced endurance (Mendoza et al., 2014). In addition, most participants in our study terminated exercise due to leg fatigue. Another potential explanation involves elevated skeletal muscle afferent signalling. Group III/IV afferent reflexes and the CB chemoreflex appear to interact in a synergistic manner, as coactivation of the reflexes elicits a greater ventilatory response than the sum of each reflex activated in isolation (for review see Oliveira et al., 2024). Supposedly both the metabo‐ and mechanoreflex components of the muscle reflex would increase with higher intensity exercise (Kaufman & Forster, 1996), and therefore heightened activity of either would increase the ventilatory response, masking the effect of CB inhibition. Previous findings from our group indicated that muscle metaboreflex activity is not increased in ILD (Chen et al., 2022), raising the question whether an aberrant mechanoreflex may be responsible.

### 4.3 Haemodynamic effects

Low‐dose dopamine in this study was associated with a decrease in BP. This is most likely due to increased vascular conductance, as evidenced by the consistent reduction in diastolic BP. While we cannot exclude the possibility of reduced cardiac output with dopamine, the preserved systolic BP and O_2_ pulse (as surrogate for stroke volume) at peak exercise (Table 4) indicates this is less likely. CB afferents modulate the sympathetic vascular outflow during exercise by contributing to the sympathetic restraint of skeletal muscle blood flow (Stickland et al., 2011, 2007). More importantly, in chronic disease associated with high CB chemoreflex sensitivity, there is abnormal contribution of tonic CB activity, evidenced by dopamine‐induced reduction in resting systematic vascular resistance in disease but not in healthy controls (Edgell et al., 2015; Phillips et al., 2018; Stickland et al., 2011). The alternative mechanism for the reduction in BP is direct stimulation of peripheral dopamine‐1 receptors by intravenous dopamine, primarily resulting in renal and mesenteric vasodilatation (Marino, 2014). This has been associated with hypotension in critically ill patients (Davis et al., 1982; Varriale & Mossavi, 1997). In the absence of muscle sympathetic nerve activity and serum catecholamine measures, it is not possible to determine the relative contribution of the two mechanisms. Furthermore, the magnitude of the reduction in BP is substantially greater than previously reported studies that used the same dose of dopamine (Collins et al., 2020; Phillips et al., 2019). The observation that the BP reduction was more prominent during exercise could be taken as evidence that CB inhibition was the major factor. A tentative interpretation is that the augmented response could reflect higher baseline sympathetic‐mediated vasoconstrictor outflow, which would be in keeping with studies showing exercise in ILD is associated with excessive sympathetic nervous activity (Boutou et al., 2021; Vaddoriya et al., 2024). Nevertheless, it is difficult to argue against a significant contribution of direct vasodilatation, although the reason(s) for the exaggerated effect is not clear. Given the effect of reduced systemic vascular resistance on the integrated cardiovascular response to exercise is complex and highly dependent on the individual's baseline physiology and degree of vasodilatation, it is perhaps not surprising that an improvement in EET was not seen.

During exercise, dopamine was also associated with heightened HR response compared to saline. This is most likely a consequence of baroreflex‐mediated compensatory tachycardia responding to the fall in arterial pressure. Direct β‐1 adrenergic stimulation by dopamine can also increase HR, but this is typically seen at doses >3 µg/kg/min (Marino, 2014) and we would expect it to be present at rest. Furthermore, there was almost doubling of the baroreflex effectiveness index albeit not statistically significant (*P* = 0.099) (Table 3). The implications of these findings for the interpretation of the results are two‐fold. Firstly, iatrogenic hypotension and compensatory tachycardia introduce an ‘artificial’ limiting factor for exercise tolerance, the effect of which would be dependent on the individual's baseline cardiac function. We note that the peak HR during the dopamine constant WR CPET was higher than that reached during the incremental CPET, indicative of cardiac limitation to exercise. Secondly, the activation of the baroreflex may confound the interpretation of CB chemoreflex activity (Boyes et al., 2024). There is evidence that baroreflex activation attenuates the sympathetic nervous system response to hypoxia (Somers et al., 1991).

### 4.4 Limitations

As previously mentioned, there is substantial variability in the dose‐specific effect of dopamine on CB sensitivity. However, determination of the optimal individual dosing was not feasible, from both a resource and a tolerability point of view. A higher dose while potentially producing higher CB inhibition, is likely to lead to vasodilatation and/or adrenergic stimulation. Although we observed a correlation between changes in CB chemosensitivity and EET, we recognise that other factors (e.g. obesity) can affect CB activity and may represent a confounding factor in this study. However, due to the small sample size and non‐normal distribution of residuals, multiple regression analysis was unable to be performed. We note the peak incremental CPET responses (Table 1) fell short of maximal effort criteria (American Thoracic Society & American College of Chest Physicians, 2003), indicating some participants may not have performed maximal exercise. We could not justify repeated incremental CPETs from a patient safety perspective. Nevertheless, a submaximal test leads to underestimation of the constant WR CPET workloads. As the relationship between WR and EET is hyperbolic, this can contribute to excessive prolongation of EETs. Longer tests are more likely to introduce unwanted factors (e.g. boredom, hip discomfort) to exercise limitation and can increase the individual variability in EET, which raises the possibility of a type 2 error (Puente‐Maestu et al., 2016). Lastly, we acknowledge that our participants were taking vasoactive prescription medications that could potentially alter the CB chemoreflex activity (Li et al., 2006) and/or vascular responsiveness. While the blood concentration half‐life of these medications is known, the duration of effect on the measured variables is less clear. Therefore, we elected against temporary cessation, which is consistent with other chronic disease studies using low‐dose dopamine (Collins et al., 2020; Phillips et al., 2019).

### 4.5 Conclusion

Low‐dose dopamine (2 µg/kg/min), administered to inhibit the CB chemoreflex, neither increased ventilatory efficiency nor led to improvements in dyspnoea and EET during loaded exercise in FILD. However, among participants displaying a reduction in CB sensitivity with dopamine, there was a strong positive correlation between the magnitude of reduction in sensitivity and improvement in EET. Ventilation was reduced by dopamine during unloaded exercise. Low‐dose dopamine was associated with larger than anticipated reduction in BP, which while suggestive of abnormal CB modulation of vasoconstrictor outflow, may also have impacted on the interpretation of our findings. Additional work is necessary to confirm this observation and establish its underlying mechanisms and potential implications for exercise tolerance and dyspnoea.

## AUTHOR CONTRIBUTIONS

Experiments were performed in the Human Cardiorespiratory Physiology Laboratory, Auckland City Hospital, Te Whatu Ora‐ Health New Zealand Te Toka Tumai Auckland. Charlotte Che: conception and design of the work, acquisition, analysis and interpretation of data, drafting of the work. Jui‐Lin Fan, Michael Plunkett and Thalia Babbage: acquisition, analysis or interpretation of data. John Kolbe: conception and design of the work. Julian F. R. Paton: conception and design of the work, interpretation of data. James P. Fisher: conception and design of the work, interpretation of data. All authors reviewed the work critically for important intellectual content, gave approval of the version to be published and agree to be accountable for all aspects of the work in ensuring that questions related to the accuracy or integrity of any part of the work are appropriately investigated and resolved. All persons designated as authors qualify for authorship, and all those who qualify for authorship are listed.

## CONFLICT OF INTEREST

None declared.

## Data Availability

Data is available upon reasonable request to the corresponding author.

## APPENDIX A

### A.1

**TABLE A1:** Medical conditions (excluding FILD‐related conditions) of participants.

Condition type	Number of patients
Hypertension	3
Previous cancer	1
Dyslipidaemia	4
Gastroesophageal reflux disease	4
Allergic rhinitis	2
Gout	2
Diabetes	1

**TABLE A2:** Long‐term prescription medication of participants.

Medication type	Number of patients
Non‐corticosteroid immunosuppressant	6
Corticosteroid	2
Angiotensin receptor blocker	3
Alpha‐blocker	1
Beta‐blocker	1
Anti‐reflux	3
Statin	3
Anti‐fibrotic	2
Bone‐protection agents	2
Anti‐hyperuricaemia agents	2
Other*	3

* *n* = 1 aspirin, *n* = 1 sildenafil, *n* = 1 diabetes medications.
